# Learning styles or study approaches in medical schools: a study of a pebble thrown into the water

**DOI:** 10.1186/s12909-025-07818-z

**Published:** 2025-09-01

**Authors:** Vildan Özeke, Işıl İrem Budakoğlu, Özlem Coşkun, Gökhan Akçapınar

**Affiliations:** 1 Curriculum & Instruction, Tokat Gaziosmanpasa University, Tokat, Turkey; 2 Medical Education and Informatics, Gazi University, Ankara, Turkey; 3 Department of Computer Education and Instructional Technology, Hacettepe University, Ankara, Turkey

**Keywords:** Learning styles, Study approaches, Medical undergraduates, Cluster analysis, k-Means algorithm

## Abstract

**Background:**

This study investigates the relationships between medical students’ learning styles and their study approaches. While learning styles have been criticized and debated, study approaches are recognized for their association with academic success. Understanding these dynamics can enhance educational practices in medical education by catering to diverse student needs.

**Methods:**

The study employs a descriptive research model with 1724 medical students using the VARK questionnaire and ASSIST inventory. We applied cluster analysis with the k-means algorithm to group students based on their learning styles. The Chi-square test of independence was used to explore the association between learning styles and study approaches.

**Results:**

Kinesthetic and Aural styles were most common, followed by Read-Write. Surface-apathetic was the most frequent study approach, followed by strategic and deep approaches. Multimodal learners showed a strong positive association with the deep approach and a negative one with the strategic approach. Kinesthetic learners also favored the deep approach, while Read-Write learners aligned with the strategic approach.

**Conclusions:**

The findings underscore the importance of recognizing the interaction between medical students’ learning style preferences and their adopted study approaches. These results can guide medical educators in designing more diverse and student-centered pedagogical strategies. Overall, the study highlights the value of promoting learners’ metacognitive awareness and fostering the development of flexible learning strategies rather than rigidly aligning instruction with specific learning styles.

## Introduction

Medical schools employ diverse instructional methods, immersing students in varied learning environments such as large-group lectures, seminars, small-group sessions, and laboratory or clinical internships. Across these settings, students encounter a range of teaching strategies delivered by healthcare professionals with different instructional styles. This diversity is crucial, because medical education encompasses a broad array of learning demands: acquiring physical skills for examinations and procedures; mastering complex, specialized knowledge; engaging with multidimensional core disciplines; and retaining detailed information from imaging, diagnostics, and pathology. Moreover, competencies such as communication, research, empathy, and professionalism are vital for effective patient care [1].

In recent decades, the shift from didactic instruction to interactive, problem-based, and student-centered learning —supported by advances in information technology— has encouraged students to manage their own learning and adopt context-specific strategies tailored to various topics and assessments [2–4]. Since the 1970 s, extensive research on student learning in higher education has led to the development of influential theoretical frameworks [5–8]. Among the earliest contributions was Marton and Saljo’s (1984) work, which introduced the concepts of deep and surface learning approaches. Subsequent studies explored individual learning preferences and strategies using self-report instruments, such as Kolb’s Learning Style Inventory [9], Biggs’s Study Process Questionnaire [6], Tait, Entwistle, and McCune’s Approaches to Learning [10], and Vermunt’s Learning Patterns [8]. These foundational studies have shaped our understanding of cognitive processes in learning [9], the quality of learning in higher education [7, 11], and the regulation of effective learning outcomes [8].

This study focuses on two constructs that have generated considerable discussion in educational research: learning styles and study approaches. Interest in these areas originally emerged from a broader concern with individual differences in learning [12]. However, the terminology surrounding these constructs is often conflated, with some studies treating learning styles and approaches as part of a single conceptual framework [12, 13].

While learning approaches describe the dynamic interaction between the learner and a given task —emphasizing contextual and affective influences —learning styles are typically viewed as relatively stable individual traits that shape how a person generally prefers to learn [14]. In this distinction, learning styles are often associated with personality-linked characteristics, whereas study approaches are seen as situational preferences that may shift depending on context.

Among the frameworks developed to assess learning styles, two have gained prominence in medical and higher education: the VARK Learning Styles Inventory [15] and Kolb’s Learning Style Inventory [9], as noted by Stirling [16] and Hernandez et al. [3]. In contrast, study approaches refer to the underlying motivations and intentions that guide students’ engagement with learning tasks [6, 7, 17]. Initially conceptualized as deep and surface approaches [5], the strategic approach was later added to capture performance-oriented learning behaviors.

Several instruments have been developed to assess study approaches. Some, such as Biggs’ Study Process Questionnaire [6], and the Experiences of Teaching and Learning Questionnaire [18–20] —were designed for course-specific contexts [21]. Among these, the Approaches and Study Skills Inventory for Students (ASSIST) [10] is the most widely used tool for evaluating students’ approaches at a general level [22].

Given these conceptual distinctions, the interplay between learning styles and study approaches in medical education remains an area of active scholarly interest. This study seeks to explore how these constructs relate to one another using well-established measurement instruments.

The following section begins by introducing the concept of learning styles, followed by a discussion of the major criticisms surrounding their use. Subsequently, the concept of study approaches is presented. Finally, a brief review of studies examining the intersection of these two constructs is provided, leading to the statement of the current study’s research aims and sub-questions.

### Learning styles

Learning style (LS) is defined as the features, preferences, choices, and ways in which students prefer receiving, processing [17], and retaining information [23] in a learning environment. LSs were analyzed by organizing them into three strata as follows [12]: (a) Cognitive personality, which includes a student’s established learning habits that happen independently of their settings; (b) information processing, which defines the learner’s approach to unfamiliar content; and (c) instructional format preference, an environmental factor which reflects the setting a student chooses for learning. Vermunt (1998) employs the term “learning style”, later called as “learning patterns”, which are broader categories of how students prefer to engage with learning [8, 24]. Vermunt proposed four main LSs: meaning-directed learners, application-directed learners, reproduction-directed learners, and undirected learners. LSs have been assessed using various tools in the current literature. In this study, we used the VARK scale primarily used in medical education to collect data. While categorizing types of learners, Boland and Amonoo [25] categorized VARK under neurocognitive approaches, which consider how our senses assimilate information.

VARK stands for the initials of the four modalities: visual, Aural, Read-Write, and Kinesthetic. An individual identified as a Visual learner prefers to learn with visual stimuli [26], a Kinesthetic learner prefers learning by doing, an Aural learner prefers to receive input by sound [26], and individuals who prefer the Read-Write modality are text-based [15]. Those whose modality scores are close to each other and do not have a specific modality that stands out are defined as Multimodal learners.

### Criticism of learning styles

The status of LSs as fixed or variable traits remains debated, with numerous factors—such as age [27], discipline [28–30], institutional culture [29], course demands [22, 28, 30], subject matter, instructional style, assessment methods [23, 29, 31, 32], prior learning experiences [29, 32], and task nature [32]—influencing them. Several studies [26, 30, 33, 34] have strongly criticized LSs, even labeling them a “neuromyth” due to insufficient empirical support and issues in both identifying LSs and applying them instructionally [17]. According to Newton & Miah, learning preferences, teaching methods, and students’ reflections on how they learn are irrelevant for determining each LS using particular classifications [34]. Another criticism is about the “meshing/matching hypothesis,” which refers to matching teaching to LSs or designing teaching methods according to LSs [1, 17, 33, 34] in healthcare education. Critics claim that determining students’ LSs and planning instruction accordingly does not benefit students [17, 26, 34, 35].

However, teaching methodology is not the only variable that affects LS, and many factors affecting learning make selecting an appropriate LS complex [13, 24]. Continued use may lead to misclassification, wasted resources, and unrealistic expectations [30, 33, 34]. Despite these criticisms, some researchers maintain that style awareness may still positively influence learning outcomes [22], and the topic remains under active investigation.

### Study skills and approaches

Study approaches refer to students’ intentions to study and learn and the learning processes they apply to achieve their goals [7, 11]. Marton and Saljo’s (1984) deep and surface approaches were replaced with “approaches to learning” or “learning approaches” in later studies. Entwistle’s approaches and study skills inventory for students [11] avoids ‘styles’ in favor of ‘strategies,’ ‘approaches,’ and ‘orientation to studying’ [7, 24, 36]. In the remainder of this article, the abbreviated use of “study approaches” is preferred instead of “study skills and approaches.”

Entwistle [11] does not suggest that these approaches are innate. Hence, these approaches are neither constant nor biological; instead, they are dynamic, and learners choose one in accordance with the situation [26, 32] and their motivation [25]. In this multifaceted view of instruction [29], contextual factors (the topic, the course design, the assessment, the teacher) and situations affect learning behaviors [21, 31, 37]. Entwistle defines contexts as the range from the broadest (the overall educational setting within the institution) to the narrowest (the content of a specific task) [7]. In brief, learning happens differently in different learning environments (regular campus-based or distance education) [24]. The learner can prefer a deep or surface-apathetic approach, and it is possible to combine several strategies [24, 32].

The “deep approach” refers to the learning process focused on understanding and internalizing a subject’s underlying concepts and principles [31]. It is characterized by the ability to think critically, apply knowledge in new situations, and connect different pieces of information [38]. This type of learning typically involves active engagement with the material and focusing on understanding the “big picture” rather than just memorizing facts [36, 39]. On the other hand, the “surface-apathetic approach” focuses on memorizing facts and information without a deep understanding of the underlying concepts or principles [38]. It is a more passive form of learning that relies on rote memorization and repetition to retain information [36]. The “strategic approach” focuses on achieving and improving grades [28]. Depending on the tasks, a strategic approach can involve deep and surface-apathetic approaches [31]. The learner aims to succeed and uses various strategies to achieve this [36]. This approach, called “organized studying,” pertains to students’ everyday study habits regarding how they structure their studies and manage their time. It is thus regarded as more of a study approach than a learning approach [40]. While categorizing types of learners, Boland & Amonoo [25] put this tripartite model [17, 25] under metacognitive approaches, which consider the role of learners’ motivations and strategies in the learning process.

Vermunt [8] have adressed strong interrelations among the learning components. The mutual coherence of LSs and study approaches is as follows: The meaning-directed and reproduction-directed styles encompass Entwistle’s [7] meaning and reproducing orientation, and Biggs’ [6] and Tait & Entwistle’s [10] deep and surface-apathetic approaches. The undirected style is similar to Tait & Entwistle’s [10] surface-apathetic approach. While Vermunt [8, 24] focuses on LSs, Entwistle’s work primarily deals with the approaches students take toward learning [7, 10]. Vermunt’s framework, focuses on cognitive, metacognitive, and affective dimensions [8, 24], is much broader than Entwistle’s approach. Entwistle’s study approaches emphasize the depth and nature of engagement with learning material [7, 10], whereas Vermunt’s LSs framework provides a more comprehensive model that accounts for a wider range of learner characteristics, including emotional and metacognitive dimensions.

### A modest contribution with potential ripple effects

Research on the interrelation between learning styles (LSs) and study approaches serves key purposes across three domains: teaching and instruction, academic performance, and cognitive and educational psychology. Understanding how LSs interact with study approaches can help optimize teaching strategies, encouraging educators to act as facilitators rather than mere transmitters of knowledge.

Such insights inform the design of inclusive curricula and instructional materials that accommodate diverse LSs, thereby promoting equity in education. Furthermore, investigating LS- study approach interactions can advance personalized learning, improve student outcomes, and align with 21st-century educational models, including adaptive learning systems and smart environments. Person-oriented approach [39], evident in adaptive learning technologies, emphasize supporting students as autonomous learners.

Promoting lifelong, self-directed learning [19] requires enabling students to recognize and apply their own LSs and study strategies. Developing metacognitive awareness and self-regulated learning, —both critical to educational success —should therefore be a priority for targeted interventions [13]. This may help students develop flexible, individualized learning pathways [41]. From a psychological perspective, these interrelations offer valuable insights into cognitive processes and learning behaviors. As learners gain awareness of their own patterns, they foster adaptability and resilience across varied contexts [42].

In sum, this line of research is essential for developing responsive and effective educational practices. This study aims to make a modest yet purposeful contribution—much like a pebble cast into still water—with the expectation that its ripple effects will extend into future research, pedagogical innovation, and the broader pursuit of adaptive, learner-centered medical education.

### The present study

Given the criticisms of LSs and the evidence supporting study approaches, our research examines whether study approaches significantly differ across LS categories. Using instruments widely employed in medical education, this study addresses the following research questions:

1. How are medical students distributed according to (a) their preferred learning styles and (b) the study approaches they adopt?
2. Is there a significant association between medical students’ preferred learning styles and their study approaches?

By addressing these questions, this study seeks to contribute to the broader understanding of individualized learning in medical education and its implications for effective educational practices.

## Method

### Research model

This study aims to determine the population’s characteristics by using the descriptive survey method from a quantitative research perspective. We aimed to reveal the interrelations among medical students’ study approaches and learning styles by using two commonly preferred instruments.

### Participants

Medical undergraduate education in Turkey is six years. The first three years are the preclinical part, and the last three years are the clinical part. Data were collected with the participation of 1724 medical students from a state university in Turkey. There are 2831 students in total, with a 61% return rate. Data collection tools were delivered to all students without using any sampling method. While 910 (52.8%) of the students are female, 795 (46.1%) are male. Nineteen individuals (1.1%) did not specify gender. The average age of the students is 20.58 (SD = 2.147), and their distribution by grade is as follows: 420 1 st year (24.4%), 374 2nd year (21.7%), 335 3rd year (19.4%), 225 4th year (13.1%), 284 5th year (16.5%) and, 86 6th year (5%).

### Data collection tools

Two data collection tools that were adapted to Turkish were used in this study. The details of the measurement tools are presented below. Data were collected using paper-and-pencil-based forms. Data collection forms were distributed to the students after they were informed. The distributed forms were delivered to the student leaders of each group, and the student leaders delivered the completed forms to the researchers. The ethical approval was given by the Clinical Research Ethics Committee at Gazi University with informed consent from all subjects (code:2017 − 307).

#### ASSIST: approaches and study skills inventory for students

Approaches and Study Skills Inventory for Students (ASSIST) was developed by Entwistle, Tait, and McCune [43] to measure students’ study skills and approaches. It was adapted into Turkish by Authors [44]. The adapted scale is divided into 12 sub-dimensions under three main dimensions (deep, strategic, and superficial). There are 14 items in the deep approach sub-dimension, 20 in the strategic approach sub-dimension, and 10 in the surface-apathetic approach sub-dimension. While Cronbach’s α reliability coefficient was 0.85 in the deep approach dimension, it was calculated as 0.89 in both strategic and surface-apathetic approach dimensions.

#### VARK questionnaire: visual, aural, Read-Write, and kinesthetic

Although it has been considerably updated over the years, the first version of the VARK Questionnaire was created by Neil Fleming in 1987 [15]. The validity and reliability study of the English version was carried out by Leite, Svinicki, and Shi [45]. The Turkish translation of the tool was made by Kalkan [46], and the version used in this study is the VARK questionnaire, which consists of 16 items. Duzgun performed a validity and reliability study of the Turkish version [47]. It helps to measure the LSs preferred by learners, such as Visual, Aural, Read-Write Kinesthetic, and Multimodal. The Cronbach’s α reliability coefficient for the total scale was 0.76.

### Data analysis

In order to group similar students to answer the first research question, the clustering method was used. The aim of the clustering is to group students who obtained similar scores according to the VARK LSs inventory. Cluster analysis was performed using Altair AI Studio data mining software with the k-Means algorithm. The goal of the k-Means algorithm is to cluster a set of n objects (here, students) into predefined k groups by minimizing variability within clusters and maximizing variability between clusters [48]. k-Means clustering was applied to a group of students based on their VARK profiles. The number of clusters (k) was manually set to five, informed by the theoretical structure of the VARK model, which comprises four primary learning styles—Visual, Aural, Read/Write, and Kinesthetic—and an additional category for multimodal learners [15]. Multimodal learners are those who exhibit strong preferences for two or more learning styles. To capture this distinct group, we specified five clusters in the k-means algorithm, anticipating that multimodal profiles (e.g., bimodal, trimodal, and quad modal) would be grouped together into a distinct cluster. This theory-driven approach ensured interpretability and alignment with the VARK framework. To verify this theory-driven clustering approach, we conducted a one-way ANOVA.

Although data-driven methods such as the elbow method or silhouette scores are commonly used to determine the optimal k, our choice was informed by theoretical and interpretive considerations grounded in the VARK model. This approach allowed for clearer alignment between cluster results and established educational constructs, facilitating both meaningful interpretation and practical relevance. 

**Fig. 1:**
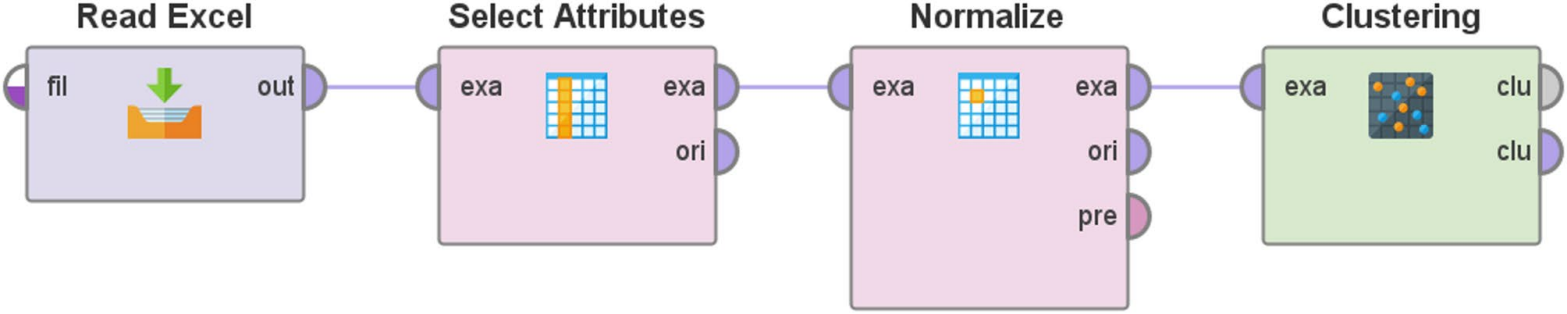
Cluster analysis process

The clustering process is shown in Fig. 1. First, data is read from the analysis file using the Read Excel operator. Then, the features used in cluster analysis are selected (see Table 1) via the Select Attributes operator. These features are normalized using z-transformation through the Normalize operator to ensure comparability across different scales. Finally, clustering is performed using the k-Means algorithm.

In the final stage, the clustering algorithm is applied. A cluster label for each student is written on the analysis file for further investigation. Since the cluster number is given as predefined (k = 5) based on the VARK scale, differences between obtained clusters were tested using ANOVA and post hoc analysis.

**Table 1:** Features used in cluster analysis

Feature Used (U) & Obtained (O)	Description
V (U & O)	Students’ total score of items related to the Visual subscale score of the students
A (U & O)	Students’ total score of items related to the Aural subscale score of the students
R (U & O)	Students’ total score of items related to the Read-Write subscale score of the students
K (U & O)	Students’ total score of items related to the Kinesthetic subscale score of the students
MM (O)	Students’ total score of items related to bimodal, trimodal, or quad modal scores of the students

As the variables are categorical, the Chi-square test of independence was used to answer the second research question. Then, post hoc analysis was conducted using R software to investigate specific differences among subgroups. Bonferroni corrections were applied to reduce Type I errors during post hoc analysis. The assumptions for the Chi-square test were also checked and met. Specifically, the sample size was adequate to satisfy the expected frequency condition: fewer than 20% of the expected cell counts were below 5, and none were below 1.

## Results

### RQ1: distribution of medical students by preferred learning styles and adopted study approaches

**Fig. 2:**
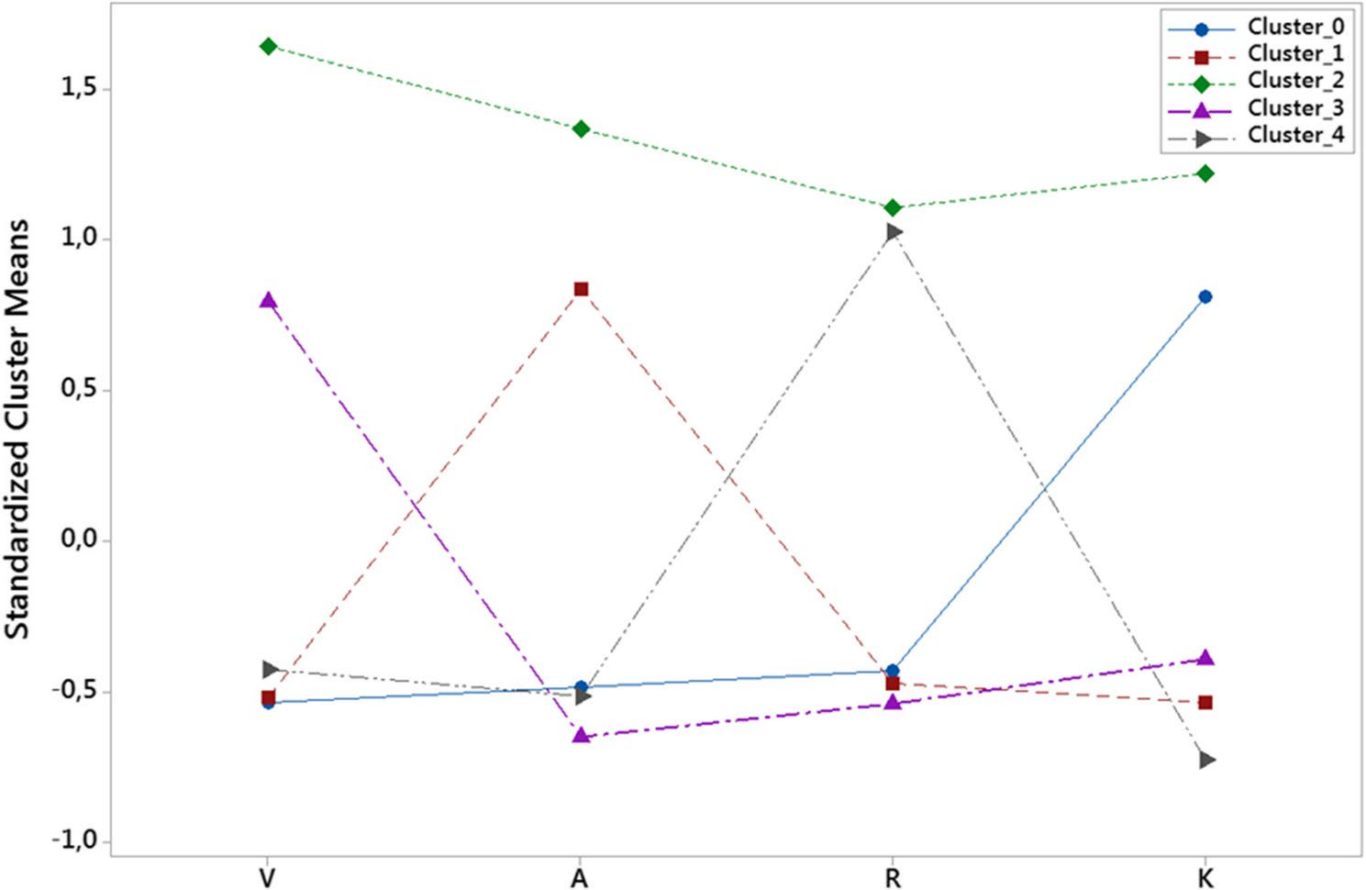
Standardized cluster means for learning style preferences

To answer the first research question, the scores students obtained from the VARK and ASSIST scales were used. Cluster analysis was employed to analyze the scores obtained from the VARK scale. The obtained clusters (Cluster_0, Cluster_1, Cluster_2, Cluster_3, and Cluster_4) were named based on the profiles of students within each cluster. Figure 2 displays the standardized cluster means for each VARK component. Each line represents a distinct cluster identified through the clustering analysis, and the symbols on the lines indicate the average standardized scores of students in that cluster for the corresponding VARK component (Visual, Aural, Read/Write, Kinesthetic). According to the graphical representation of cluster means given in Fig. 2, the clusters were named as follows: Cluster_0 as kinesthetic learners, Cluster_1 as aural learners, Cluster_2 as multimodal learners, Cluster_3 as visual learners, and Cluster_4 as read-write learners.

An ANOVA analysis was conducted to test whether there is a significant difference between the mean scores obtained from the scale’s sub-dimensions by students in different clusters. The results in Table 2 demonstrate statistically significant differences between clusters.

**Table 2:** Descriptive statistics and ANOVA results

	Cluster_0 (K) (*N* = 441)	Cluster_1 (A) (*N* = 407)	Cluster_2 (MM) (*N* = 197)	Cluster_3 (V) (*N* = 339)	Cluster_4 (*R*) (*N* = 340)	F (df = 4, 1719)	η^2^
Visual	2.46 ± 1.20	2.50 ± 1.36	7.65 ± 2.41	5.63 ± 1.41	2.73 ± 1.37	662.88***	0.60
Aural	4.33 ± 1.55	7.42 ± 1.27	8.65 ± 2.29	3.95 ± 1.37	4.26 ± 1.35	581.99***	0.57
Read-Write	3.61 ± 1.44	3.52 ± 1.37	6.88 ± 2.30	3.37 ± 1.26	6.71 ± 1.36	438.46***	0.50
Kinesthetic	7.29 ± 1.35	4.23 ± 1.45	8.22 ± 2.30	4.55 ± 1.52	3.79 ± 1.35	505.84***	0.54

*** *p* <.001

The distribution of students based on the clusters representing their learning styles, as identified through cluster analysis, is as follows: 441 students (25.6%) exhibited a kinesthetic learning preference, followed by 407 (23.6%) with an aural preference, 340 (19.7%) with a read/write preference, and 339 (19.7%) with a visual preference. Additionally, 197 students (11.4%) demonstrated a preference for multimodal learning. On the other hand, in terms of study approaches, 654 students (37.9%) adopted a surface-apathetic approach, 543 (31.5%) followed a strategic approach, and 527 (30.6%) employed a deep approach.

###  RQ2: the relationship between learning styles and study approaches

The interrelations among students’ preferred LSs and study approaches (based on ASSIST scale scores) have been investigated within the scope of the second research problem. The results of the Chi-Squared test of independence (5 × 3) meaningful insights into the relationship between LSs (VARK) and study approaches (ASSIST) (χ^2^_(8,1724)_ = 37.367, *p* <.001). The results of the post-hoc analysis conducted to see which specific subgroups have an association are presented in Table 3.

**Table 3:** Contingency tables and Chi-Squared test results for learning styles and study approaches

	Learning Styles				
Study Approaches	Kinesthetic n (%) (Chi-square)	Aural n (%) (Chi-square)	Multimodal n (%) (Chi-square)	Visual n (%) (Chi-square)	Read-Write n (%) (Chi-square)
Deep Approach	160 (36.3%) (9.114)^*^	103 (25.3%) (6.948)	79 (40.1%) (9.523)^*^	90 (26.5%) (3.211)	95 (27.9%) (1.378)
Strategic Approach	124 (28.1%) (3.136)	130 (31.9%) (0.049)	40 (20.3%) (12.91)^*^	119 (35.1%) (2.544)	130 (38.2%) (8.916)^*^
Surface-Apathetic Approach	157 (35.6%) (1.371)	174 (42.8%) (5.249)	78 (39.6%) (0.26)	130 (38.3%) (0.031)	115 (33.8%) (3.042)

*Significant associations after Bonferroni correction, Adjusted *p* =.003333

**Fig. 3:**
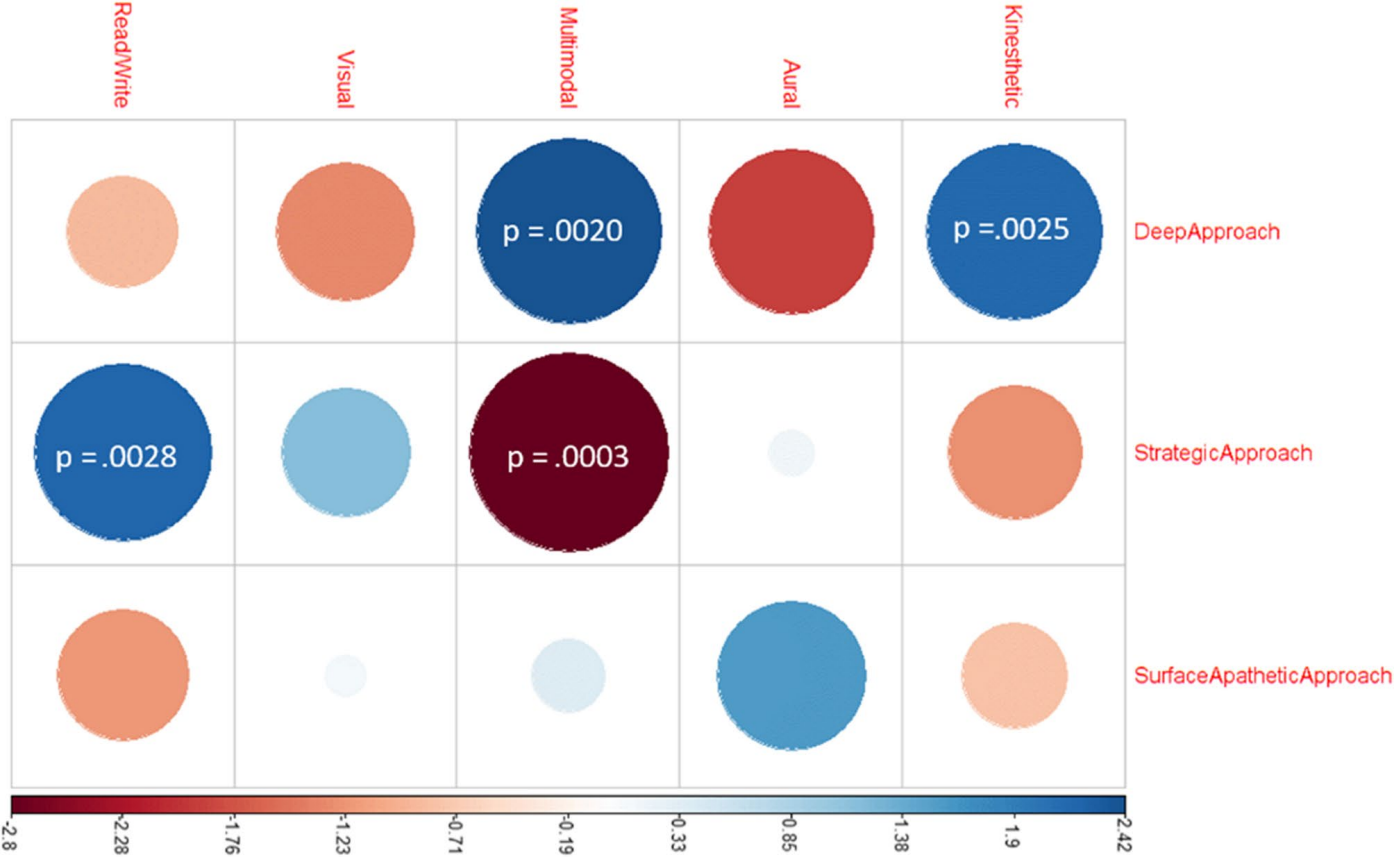
Visualized Pearson Residuals and *p*-values for statistically significant associations

As shown in Table 3 and visualized in Fig. 3, significant associations were observed between specific learning styles and study approaches among medical students. Kinesthetic learners demonstrated a strong positive association with the deep approach, suggesting that students who prefer hands-on, experiential learning are more likely to engage meaningfully with course content—an especially relevant finding in medical education, where clinical reasoning and applied knowledge are critical. Multimodal learners showed a significant negative association with the strategic approach and a significant positive association with the deep approach. This suggests that while these students benefit from flexibility in how they process information, they may require additional support in adopting goal-oriented study strategies. Read-Write learners, on the other hand, were significantly linked to the strategic approach, which reflects structured and performance-oriented learning behaviors. No significant associations were found for study approaches across visual and aural learners. Although not statistically significant, aural learners have a positive association between surface-apathetic and a negative association between deep approaches. Figure 3 visualizes these associations through Pearson residuals and p-values, clearly showing the strength and direction of each significant relationship.

These findings contribute to a better understanding of how tailoring study approaches according to students’ learning preferences can improve engagement and learning outcomes for medical educators and curriculum designers. First, educators should consider integrating active learning techniques—such as problem-based learning, simulation, or clinical skills labs—to engage kinesthetic and multimodal learners more effectively. Second, students with multimodal preferences may benefit from explicit instruction in time management and assessment-focused strategies to enhance their strategic approach, especially given the high-stakes and performance-oriented nature of medical training. Finally, by recognizing the diversity of learning styles in their classrooms, medical educators can adopt more inclusive and adaptive teaching methods that not only improve engagement but also support better academic outcomes. Together, Table 3; Fig. 3 provide complementary perspectives on these associations, enhancing the clarity and applicability of the findings within medical education.

## Discussion

This study examines a longstanding issue in medical education: the relationship between learning styles and study approaches. Drawing on a large-scale and well-structured dataset, it combines the VARK and ASSIST instruments with cluster analysis and inferential statistics to generate meaningful insights.

1. Distribution of medical students by preferred learning styles and adopted study approaches.

This study contributes to the growing body of research examining the complex relationship between learning styles and study approaches in the context of medical education. In our study, most of the students prefer the kinesthetic style. In some studies [4, 49], similar results were found for the kinesthetic style, the second preferred LS among medical students in another study [50]. The high number of practical activities and contents based on application in medical education can also be interpreted concerning this type of learning. The Aural style is the second preferred LS in our study and in Bokhari and Zafar’s [49] study. In Prithishkumar and Michael’s [51] study, the results are very similar, where the difference between aural and kinesthetics is too close, but the aural learners came first order. For the “nontraditional” medical students, aural and kinesthetic modes are believed to predominate [42]. The most preferred LS among medical students was multimodal [2, 3, 23, 27, 50, 52]. However, the number of multimodal students is the lowest in our study (11.4%).

The results related to study approaches show that medical students preferred all the approaches. In terms of study approaches, 654 (37.9%) learners adopt the surface-apathetic approach, 543 (31.5%) have a strategic approach, and 527 (30.6%) have a deep approach.

2) The relationship between learning styles and study approaches.

The association between learning styles (LSs) and study approaches has been explored in this part of the study. The results indicate that Kinesthetic and Multimodal learners tend to prefer a deep approach, while Read-Write learners are more likely to adopt a strategic approach. Notably, Multimodal learners demonstrate a significant aversion to the strategic approach, and Aural learners show an association with the surface apathetic approach. These findings have theoretical and practical implications across areas such as instructional design, student success strategies, and cognitive and educational psychology. In contrast to our results, a study conducted with accounting students reported no significant influence of LSs on study approaches [53].

Our findings are consistent with prior research suggesting that Kinesthetic learners often favor a deep approach to learning [2, 54]. This reinforces the notion that medical students benefit from hands-on, experience-based educational methods, as Kinesthetic learning typically involves multimodal sensory engagement—particularly relevant in the context of clinical and skills-based training [54].

Multimodal learners, who favor the deep approach and avoid strategic learning, tend to prioritize understanding over performance. While this promotes deeper cognitive engagement, it may create challenges in assessment-driven or highly structured academic environments. The presence of multimodal learners poses unique challenges and opportunities for medical educators. Therefore, educators might consider providing guidance to help multimodal learners apply their flexibility in more goal-oriented ways, particularly in high-stakes contexts where strategic planning is essential. In performance-oriented settings, supporting these learners requires more than simply offering content in multiple formats [55]. Rather, instructional design should incorporate metacognitive scaffolding that enables students to select and combine strategies based on the demands of specific tasks. For instance, integrating structured flexibility—such as modular content delivery, diverse assessment formats, and opportunities for self-selection in learning activities—can promote more intentional and strategic engagement. These approaches can help learners better align their study methods with contextual needs, thereby enhancing both adaptability and academic performance.

The significant association between read-write learners and the strategic approach also reflects previous findings [49], highlighting a tendency to thrive in structured learning environments with clearly defined outcomes. However, this performance-oriented mindset may sometimes limit opportunities for deeper learning. Instructional strategies could, therefore, aim to more reflective and exploratory learning behaviors in these students, while still leveraging their organized and goal-driven strengths. Aural learners’ association with the surface apathetic approach may be partially explained by the high academic workload and predominantly didactic instructional formats typical of medical curricula. Students adopting a surface-apathetic approach tend to rely on rote memorization and avoid restructuring material [36]. Aural learners may benefit from interactive strategies such as discussions, verbal repetition, and the use of mnemonic devices [56]. However, limited opportunities for student-centered dialogue in large lecture-based settings may reinforce their reliance on passive learning habits [17]. Another reason is that medical undergraduates may be primarily exposed to didactic learning in lecture halls because of the class size.

Visual learners, in contrast, did not demonstrate a significant association with any particular study approaches in this study. Although some previous research has reported a significant relationship between visual preferences and surface-apathetic approaches [51, 54], our findings did not support this association.

Although learning styles alone do not determine how students study, our findings indicate that students with different learning styles often adopt distinct study approaches. Rather than tailoring instruction rigidly to learning style categories, it may be more effective to raise students’ awareness of diverse learning strategies and promote the development of flexible study habits. Encouraging medical students to reflect on their learning behaviors and expand their strategic repertoire—regardless of dominant style—can enhance adaptability to diverse academic demands.

However, these findings must be interpreted critically in light of the theoretical limitations of the learning styles framework. Rigid categorization risks oversimplifying the complex, context-dependent nature of learning, and empirical support for the “matching hypothesis” remains limited. Accordingly, our study does not endorse fixed typologies but views learning style preferences as flexible tendencies that may shape—though not dictate—engagement strategies. By emphasizing metacognitive awareness and adaptive flexibility, we aim to contribute to a more nuanced understanding of learner diversity.

Building on these insights, the associations observed in this study emphasize the importance of equipping learners with the tools to flexibly navigate between learning strategies. While LSs may shape students’ natural inclinations toward content engagement, fostering intentional strategy use—particularly in response to context—can promote more resilient and effective learners. These findings can inform educational practices aimed at helping medical students become reflective, adaptable, and independent in managing their learning throughout their academic and professional journeys.

The inclusion of over 1700 participants from all years of a 6-year medical program gives robustness and breadth to the findings. Despite the large sample size, diverse representation of medical students, and the methodological clarity, this study has several limitations. Firstly, the data were collected through self-report instruments (VARK and ASSIST), which are subject to biases such as social desirability, selective memory, or subjective self-perception. These limitations may affect the accuracy of reported learning preferences and study behaviors. Secondly, the cross-sectional design does not allow us to track changes in students’ learning preferences and study approaches over time. Longitudinal studies could provide richer insights into how these preferences evolve during medical education. Additionally, the reliance on quantitative measures limits our understanding of the contextual and experiential factors that influence learning. Future research incorporating qualitative methods—such as interviews or focus groups—could better capture the nuances of how students perceive and adapt their learning strategies. Further studies can examine the recommended learning paths applied by different preferences of medical undergraduates and their achievements in adaptive learning environments [39]. Profiling studies with further analysis may be conducted.

## Conclusion

This study advances the literature on individualized learning in medical education by highlighting implications for curriculum design and instructional strategies. Based on cluster analysis, medical undergraduates were grouped according to their LSs and study approaches. The findings indicate that approximately one in four students demonstrated a kinesthetic preference, with a comparable proportion showing an aural learning preference. Regarding study approaches, around one-third of students were characterized by either a surface-apathetic or a strategic approach.

The analysis revealed that LSs and study approaches are not independent constructs; rather, they are significantly associated. Specifically, kinesthetic and multimodal learners were more likely to adopt a deep approach to learning. Multimodal learners tended to avoid the strategic approach, while read-write learners showed a significant inclination toward strategic study behaviors. Although, aural learners exhibited a tendency toward the surface-apathetic approach and avoidance of the deep approach, these associations did not reach statistical significance.

Although the title of this study uses “or,” the research itself adopts an integrative “and” perspective. Rather than considering learning styles (LSs) in isolation, we argue that a combined analysis of both LSs and study approaches yields a more comprehensive understanding of learners. One criticism often raised against the concept of learning styles is that LS inventories tend to capture a relatively narrow dimension of learning behavior, constrained by the theoretical framework on which they are based [13, 21, 24]. By incorporating measurement tools grounded in different theoretical models and examining the patterns and interactions between them, it is possible to gain deeper insight into the complex and dynamic nature of the learning process. Such an approach can also generate valuable data that contribute meaningfully to both cognitive psychology and educational research.

When discussing effective learning and teaching practices, it is often difficult to “sift the wheat from the chaff” [29], especially in complex educational settings like medical education. Given the diversity of learning contexts, student preferences, and assessment formats in medical training, identifying what truly supports effective learning is challenging. Our findings contribute to this discussion by highlighting patterns between learning styles and study approaches, suggesting that fostering students’ awareness of their learning habits may be a constructive step toward supporting more adaptive and reflective learning behaviors.

Overall, this study contributes to the understanding that, rather than endorsing fixed learning styles or study approaches, educational efforts should focus on equipping students with a diverse repertoire of learning strategies. Adapting to the demands of higher education requires not only sustained effort but also self-awareness and reflective engagement with one’s own learning process. This study offers a modest yet purposeful contribution—much like a pebble cast into still water—with the hope that its ripple effects will extend into future research, pedagogical innovation, and the ongoing advancement of adaptive, learner-centered medical education.

### Pedagogical implications

This study offers several pedagogical implications for medical educators aiming to address diverse learning preferences and study approaches. For kinesthetic learners, incorporating more hands-on and experiential activities [42]—such as laboratories, clinical rounds, cadaver dissections, role-playing, and simulation-based learning [4, 16] —can promote deeper engagement and foster deep learning. These environments, supported by observation, feedback, and repetition [57], allow kinesthetic learners to practice procedural skills safely and meaningfully. Multimodal learners may benefit from an integrated curriculum that offers flexibility in content delivery and assessment formats. Designing instruction that blends various sensory modalities (e.g., visual aids, spoken content, reading tasks, and physical interaction) can enhance comprehension and retention across learner types. Metacognitive prompts that guide learners in choosing appropriate strategies for a given task may further enhance the effectiveness of multimodal engagement. For read/write learners, strategic engagement may be supported through structured note-taking, essay-based assessments, and reading-intensive assignments. These activities align with their preference for text-based input and output and may reinforce organized, goal-oriented study approaches. Aural learners, particularly those who may rely more heavily on surface strategies, can benefit from instructional methods that include spoken explanations, podcasts, group discussions, and opportunities for verbal interaction. These approaches may encourage greater engagement and, when paired with reflective activities, can support deeper processing of content. Stirling [16] puts lectures, discussions, tutorials, audio records, and practicing verbal orders under the aural category.

By aligning instructional strategies with students’ dominant learning preferences—without rigidly categorizing them—educators can promote adaptable, metacognitively aware learners who are better equipped to navigate the varied demands of medical education.

## Data Availability

The datasets analysed during the current study are available from the corresponding author on reasonable request.

## Abbreviations

LS: Learning style
ASSIST: Approaches and Study Skills Inventory for Students
VARK: Visual, Aural, Read-Write, and Kinesthetic
